# Modified neuroimmune processes and emotional behaviour in weaned and late adolescent male and female mice born via caesarean section

**DOI:** 10.1038/s41598-024-80770-y

**Published:** 2024-11-30

**Authors:** Mathieu Di Miceli, Moïra Rossitto, Maud Martinat, Flore Marchaland, Sarah Kharbouche, Marion Graland, Farah Younes, Alexandra Séré, Agnès Aubert, Lydia Rabbaa Khabbaz, Charlotte Madore, Jean-Christophe Delpech, Rebeca Martín, Sophie Layé

**Affiliations:** 1 Université de Bordeaux, INRAE, Bordeaux INP, NutriNeuro, UMR 1286, Bordeaux, France; 2 Worcester Biomedical Research Group, School of Science and the Environment, University of Worcester, Henwick Grove, Worcester, WR2 6AJ UK; 3 Faculty of Biological Sciences, School of Biomedical Sciences, University of Leeds, Leeds, LS2 9JT UK; 4 Laboratoire de Pharmacologie, Pharmacie Clinique et Contrôle de Qualité des Médicaments, Faculty of Pharmacy, Saint-Joseph University of Beirut, Beirut, Lebanon; 5 Micalis Institute, AgroParisTech, INRAE, Université Paris-Saclay, 78350 Jouy-En-Josas, France

**Keywords:** C-section, Birth delivery mode, Inflammation, Behaviour, Sociability, Microglia, Neuroinflammation, Lipids, Neuroscience, Molecular neuroscience, Neuroimmunology, Social behaviour

## Abstract

Elective and emergency Caesarean section (C-section) procedures are on the rise, exceeding the recommended guidelines by the World Health Organization. Higher morbidities and long-term health conditions are correlated to C-section deliveries, including neurodevelopmental disorders. During C-section delivery, newborns are not exposed to the vaginal commensal flora, which impedes the early establishment of the gut microbiota. The latter is essential for adequate neuro-immune processes to take place during infancy. In this study, we used a validated model of mice born by C-section (CSD), which mimics clinical observations of dysregulated gut microbiota. Animals were either born naturally or by CSD, before being adopted by dams who underwent delivery within the 12 preceding hours. Behavioural analyses were conducted at post-natal day (PND) 21 and 55. Our results indicate that animals born by C-section present significantly higher body weight in late (PND40-P53) but not early adolescence (PND21-P27), compared to animals born by vaginal delivery (VD). Male animals delivered by C-section presented significantly lower exploration time of the novel arm in the Y Maze test at PND55. However, at PND21, abnormal social interaction was witnessed in male and female animals born by CSD, with significantly decreased time spent interacting during the social interaction test. At both PND21 and PND55, animals from both sexes born by C-section presented significantly decreased time spent in the open arm of the Elevated Plus Maze test, compared to control animals. We then measured the expression of genes associated to neuroimmune interactions (microglia phenotype), inflammatory mediators and lipids in several brain structures of VD and CSD mice at PND21 and PND55. At weaning, animals born by CSD presented altered microglia, inflammatory and lipid metabolism signatures, with increased expression of *Cd36*, *Csf1r* and *Tnfα* in different brain regions of males, but not in females. At PND64, *Csf1r*, *Tmem119* as well as *C3ar1* were significantly increased in males born by C-section, but not in females. In males born by vaginal delivery, the expression of *Cd36* at PND64 was correlated to anxiety at PND55, whilst a correlation between the expression of Clec7a and the number of head dippings in the elevated plus maze was also noted in males born by CSD. Altogether, our study shows altered emotional behaviour in animals delivered by CSD, which is likely explained by underlying neuro-inflammatory processes in different brain regions. Our work further supports the long-term consequences of CSD on brain health.

The global number of infants delivered by Caesarean-section (C-section) has significantly risen in recent years, surpassing the World Health Organization’s recommended guidelines of 10–15% in many countries^1,2^. Such an increased rate is particularly worrying as C-section delivery (CSD), which is a standard childbirth surgical procedure for pregnant women unable to naturally deliver^3^, has been associated with a higher risk of neonatal morbidity and long-term health conditions such as type 1 diabetes^4,5^ or obesity^6,7^. In addition, neurodevelopmental disorders (NDDs), such as autism spectrum disorder (ASD), are at higher frequency in children born by CSD^8,9^, which can be worsened by general anaesthesia and could be dependent of sex^10^. Thus, CSD may impact post-natal brain development in a sex-dependent manner, however the underlying molecular mechanisms are still poorly known.

Recent data revealed that the alteration of gut microbiota vertical transmission induced by CSD could be involved in the associated increased risk of developing NDDs in later life^11–13^. Indeed, perinatal exposure to maternal microbiota is essential for optimal timing of development, including of the immune system and of the brain^14^. Moreover, gut microbiota maturation aligns with this critical window of early brain development during the first 2–3 years of postnatal life^15,16^. A lack of maternal microbiota transmission to progeny, as observed in germ-free mice, has been repeatedly reported to alter brain development^17,18^ and impact offspring long-term health and behaviour^19–22^.

During brain development, microglia cells, the resident macrophages of the brain, highly contribute to brain shaping and synaptic pruning^23,24^. Microglia molecular phenotype is in link with their functions during life^25,26^. In addition, microglia can be strongly influenced by any environmental and inflammatory insults, even during the gestational period, thereby altering their neurodevelopmental functions, which, in turn, alter neuronal network shaping and early and later life behaviour^27–34^. In addition, recent works pinpoint that microglia functions are also altered by microbiota composition. In germ-free mice, microglia display morphological and functional differences, accompanied by increased neuronal death, which can be normalized by microbiota transfer^35–38^.

Variations in the microbiomes of CSD babies compared to vaginally delivered (VD) infants have already been reported^39,40^. CSD babies display elevated levels of hospital associated opportunistic bacteria shortly after birth and, over time, exhibit a deficiency in typical gut microbes essential for immune health^41^. C-section murine models have been previously established^42^. In such models, microbiota diversities present similar alterations to those observed in human CSD newborns^39,43^. We and others recently found alterations in T cell and inflammatory responses in the CSD model^43–45^. In addition, mice born by CSD also exhibit various neurobiological and behavioural traits akin to those observed in infants born by CSD Furthermore, CSD mice also exhibit various neurobiological and behavioural traits akin to those observed in infants delivered by C-section^46,47^. However, the impact of C-section on the trajectory of emotional and cognitive behaviours and neuroinflammatory parameters and whether this is affected in a sex-dependent manner is still poorly known. In the current study, we used the previously-developed CSD model^43^ to assess emotional and social behaviours and spatial memory of male and female mice at weaning and late adolescence. In addition, neuroinflammatory processes were evaluated in different brain areas associated with these behavioural parameters at weaning and late adolescence in male and female mice. Overall, our work reveals for the first time that C-section influences behavior and neuroinflammatory factors at weaning and late adolescence in a sex-dependent manner.

## Methods

### Animals, ethics and experimental design

**Fig. 1:**
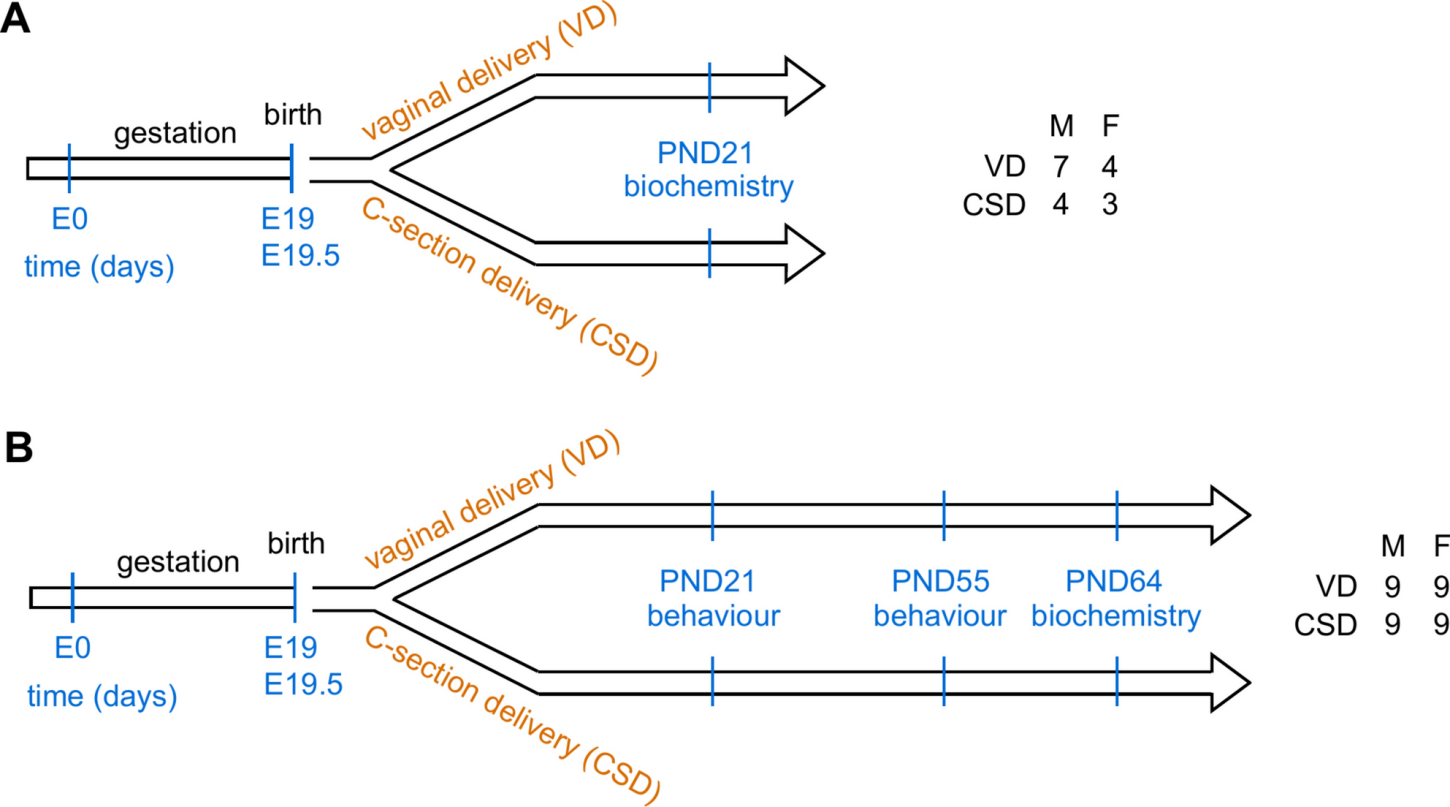
Experimental design of the current study. (**A**) The first cohort was randomly divided between E19-E21 into two experimental groups: mice delivered naturally (vaginal delivery, reference group) or mice born by C-section delivery. Both males and females were used. At weaning (PND21), biochemical analyses were performed. Numbers on the right represent number of animals in each group. (**B**) The second cohort was performed as in (**A**), but several different analyses were performed: mice underwent behavioral assessments at PND21 (i) and PND55 (ii), followed by biochemical analyses at P64 (iii). Numbers on the right represent number of animals in each group. E: embryonic day, PND: post-natal day, VD: vaginal delivery, CSD: C-section delivery.

Pregnant RjOrl:SWISS mice (CD1) were procured from Janvier (Le Genest Saint Isle, France) and were individually housed under specific pathogen-free (SPF) conditions at 21–22 °C within the animal facilities of the French National Research Institute for Agriculture, Food and Environment (IERP, INRAE Jouy-en-Josas, France). On gestational day 19, pregnant females underwent hysterectomy, and litters were transferred to mothers that had undergone vaginal delivery within the preceding 12 h (C-section Delivery, CSD group) as previously described^43^. Control mice were delivered vaginally (VD group) between gestational day 19 and 19.5. To mitigate potential biases, pregnant mice that delivered vaginally exchanged their litters. At 14 days of post-natal life (PND), mothers and pups were sent to the NutriNeuro SPF animal house (INRAE Bordeaux, France). At weaning (PND 21), male and female offspring from CSD and VD groups were randomly separated and housed in groups of 6–9 in polycarbonate cages in a room air-conditioned (22 ± 1 °C) with a 12:12 light/dark cycle. Experiments were performed on 2 different cohorts of male and female mice (Fig. 1). The first cohort was randomly divided into two different groups: (i) mice born by VD (n = 11) or (ii) mice delivered through CSD (n = 7) in which biochemical analyses were performed at PND21 (Fig. 1A). The second cohort of mice was also divided into two different groups, similarly to the first cohort (VD (n = 18) and CSD (n = 18), Fig. 1B). In this cohort, two sets of behavioral assessments were performed, one at PND21, another at PND55, before biochemical analyses at PND64. The experimental procedures are illustrated in Fig. 1.

During all the experiments, mice had ad libitum access to water and food. Conventional mice were housed in specific pathogen-free (SPF) conditions. All experiments were conducted in accordance with the criteria outlined in the European Communities Council Directive 2010/63/EU for animal experiments. Ethical approvals were obtained from the French National Ethical Committee for the Care and Use of Animals (CEEA: Comité d'Ethique en Expérimentation Animale, approval numbers: APAFIS 2018061514419181 #15533 and APAFIS 202001072155893 #23506). All experiments were approved by the French Ministry for Higher Education and Research (Ministère de l’Enseignement Supérieur et de la Recherche). The reporting in this manuscript was done in accordance with ARRIVE guidelines^48^.

### Behavioral assessments

Behavioral tests were performed on CD and VSD mice at PND21 and PND55 during the light phase (between 8:00 and 11:00 AM) as previously described^33,49,50^. A tracking system was used to analyze the different parameters for each test (SMART system; San Diego Instruments).

Social Interaction (SI) experiments involved assessing the animal’s ability to interact with an unfamiliar individual and were measured as previously described^49,51^. Mice were introduced into an open field (40 × 40 cm, 20 cm deep) with a transparent Plexiglas box positioned in one corner and illuminated at 30 Lux. The area was delineated into interaction zones, comfort zones, and a safe zone. In the initial phase of the test, the Plexiglas box was vacant, allowing the mouse to explore the area for 5 min. Subsequently, in the second phase, an unfamiliar mouse was introduced into the Plexiglas box, and the test mouse was placed back in the center of the open area for an additional 5 min of exploration. The time spent in each area, as well as the distance covered, was meticulously recorded. Furthermore, the number of interactions was quantified when the test mouse made contact with the novel mouse through the Plexiglas box.

Anxiety-like behavior was assessed using the Elevated Plus Maze (EPM) task as previously described^49,52^. This apparatus comprised an elevated structure featuring two open arms and two “closed” arms enclosed by walls. Mice were positioned in the center of the maze, and both the distance covered and time spent in different areas were meticulously recorded over a 5-min period, under a brightness of 15 Lux. The recorded parameters included the time spent in the arms and the number of head dippings, where the mouse extended its head below the maze’s height.

Spatial recognition memory was assessed using the Y-maze test, as described previously^33,53^. The Y-maze apparatus consists of three arms (35 cm long and 10 cm deep), illuminated at 15 Lux, and is situated in a room with distinct visual cues. In the initial trial, mice explored two arms for 5 min. Following a 1-h inter-trial interval (ITI), mice then explored all three arms for another 5 min. The assignment of initial and new arms was randomized for each mouse. The analysis involved comparing the time spent in the familiar and new arms to determine whether the mice recognized the new arm.

### Neuroimmune parameters

The expression of several markers characteristic of neuroinflammatory processes, such as proinflammatory cytokines (*TNFα*, *IL-1β*, *IL-6*), lipid metabolism and transport (*Cd36*, *Alox12*, *Mfsd2a*, *Fabp5*, *Ephx2*) as well as microglia phenotype (*Itgam* (*Cd11b*), *Cx3cr1*, *Cx3cl1*, *P2ry12*, *Csf1r*, *Clec7a*, *Apoe*, *Tmem119*, *Trem2*) (Supplementary Table S1) was assessed in the hippocampus (HC), prefrontal cortex (PFC) and striatum (Strm) by real-time PCR, as described previously^33^. Briefly, mice were first anesthetized with an intraperitoneal injection overdose of sodium pentobarbital at 400 mg/kg (100 µl/g). After absence of hind paw withdrawal reflex was observed, mice were then transcardially perfused with 10 ml of ice-cold phosphate-buffered saline solution (NaCl 137 mM, KCl 2.7 mM, Na_2_HPO_4_ 8 mM, KH_2_PO_4_ 2 mM, pH 7.4, 8 ml/min). Subsequently, mice were decapitated and brains were rapidly harvested and dissected out. Total RNA from brain structures was extracted using TRIzol reagent with chloroform (22,711.290, VWR) and isopropanol (20,880.320, VWR). RNA purity and concentration were assessed using a Nanodrop spectrophotometer (Nanodrop Technologies, Wilmington, DE). Then, a total of 2 µg of RNA was reverse-transcribed into cDNA using Superscript III (Invitrogen, Life Technologies), recombinant DNAase I (Invitrogen, Life Technologies) and random primers according to the manufacturer’s protocol. Quantitative PCR was carried out on 384-well plates using LightCycler 480 Instrument II (Roche). We used LightCycler 480 Probes Master (Roche) for TaqMan technology, which used 10 µl of cDNA diluted 1:5 (20 ng/µl) and amplified by real-time PCR. We also used TB Green Premix Ex Taq II (Takara Bio) for SYBR Green technology, which used 10 µl of cDNA diluted 1:40 (2.5 ng/µl) and amplified by real-time PCR. Gene expression was normalized to beta-2-microglubulin (*B2m*), a housekeeping gene, using the delta-CT method (2^−ΔΔCT^). Primer sequences are detailed in Supplementary Table S2.

### Data analysis

Data analysis and representation were performed in RStudio v 4.0.3^54^ using base functions as well as functions included in the following packages: *psy*, *corrplot*, *ggcorrplot*, *heatmap*.*plus*, *heatmap3*, *ggplot2*, *ggbiplot*, *Hmisc*. These are available open-access at CRAN (Comprehensive R Archive Network): https://cran.r-project.org/. Two-way repeated measures analysis of variations (ANOVAs) were used when 1 variable depended upon 2 variables^55^, without assumption of sphericity. Following significant ANOVA, Šidák’s post-hoc tests were used^56^. Normality was assessed using Shapiro–Wilk tests^57^. To compare 2 groups, normally-distributed data were analyzed with Welch’s t-tests^58^, whilst Mann–Whitney U tests were used when data was not normally-distributed^59^. When data was paired and normally-distributed, paired t-tests were used whilst Wilcoxon tests were used when data was paired and non-normally-distributed^60^. A probability value below 0.05 (*p* < 0.05) was considered significant. Please refer to Supplementary Table S3 for additional information on data analysis. To analyze large-scale behavioral data, principal component analysis (PCA) was performed using scaled data, centered around median values. Data capture by the PCA analysis was assessed on the first two principal components. Since our large scale data involved experimental results in 28 variables (cohorts 1 and 2, presented in Supplementary Table S4), some limitations were thus introduced, often referred to as the ‘curse of dimensionality’^61^, although it was rather described as ‘a blessing’ more recently^62^. Pearson’s correlation coefficients were displayed on matrices and were calculated with pair-wise complete observations. Please refer to Supplementary Table S3 for statistical analyses and statistical results.

## Results and discussion

### Social impairments induced by CSD

We examined spatial memory and emotional behavior in mice delivered through VD or CSD at both weaning and late adolescence. Behavioral evaluations were consistently conducted within the same cohort of animals. Notably, male mice subjected to CSD exhibited increased body weight in late adolescence (Supplementary Fig. 1), consistent with previous findings^63^. However, such weight differences were not observed amongst female mice.

**Fig. 2:**
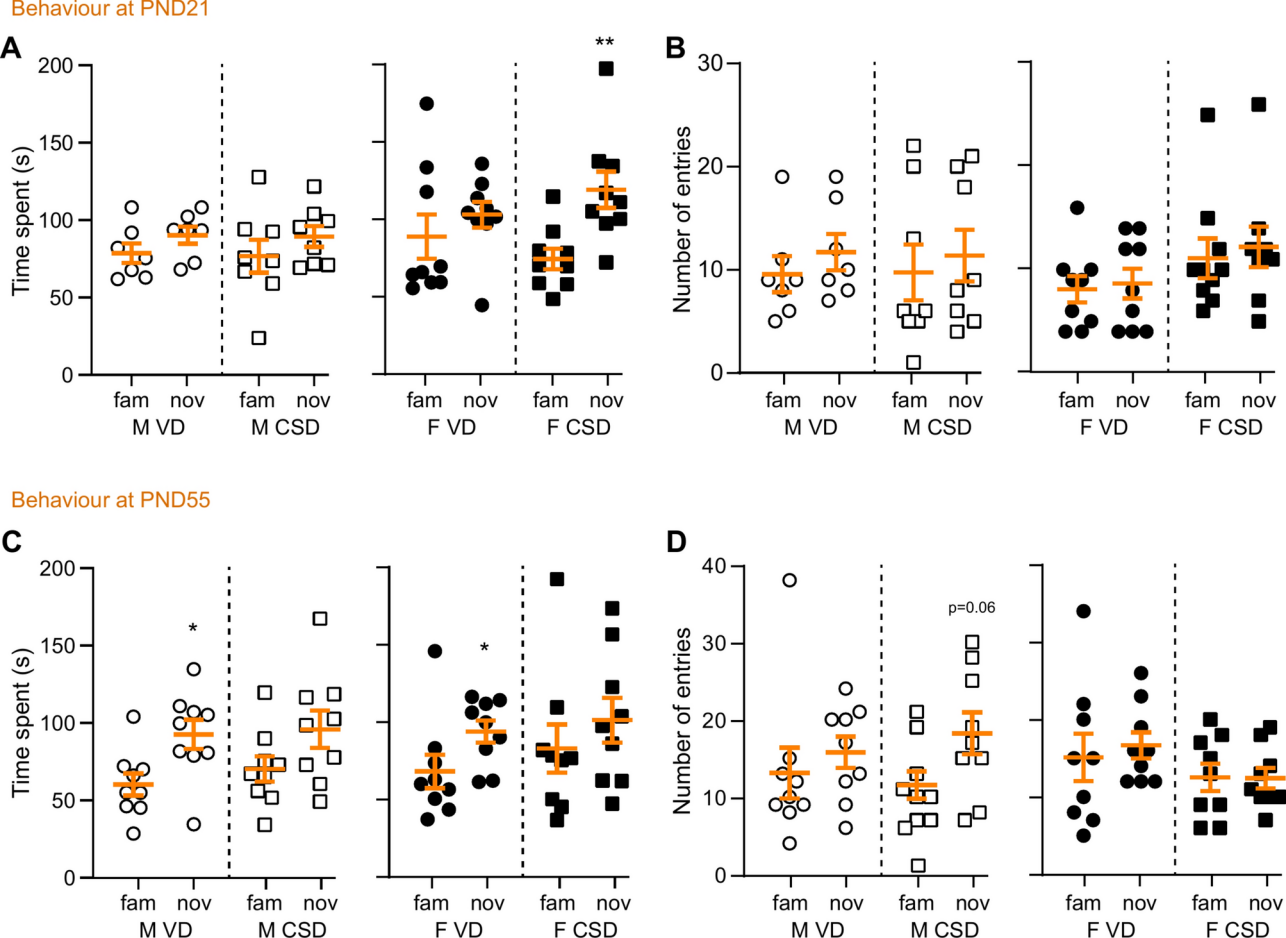
Behavioral assessments at PND21 and PND55 in the Y Maze. (**A**) Time spent in the familiar (fam) or novel (nov) arms at PND21. **p < 0.01, Welsh unpaired t-test. (**B**) Number of entries. (**C**, **D**) Similar assessments at PND55. *p < 0.05, Mann–Whitney test. M: males, F: females, VD: vaginal delivery, CSD: C-section delivery.

To delve into spatial memory, we employed the Y-maze test at PND21 and PND55 (Fig. 1). At weaning, none of the male mice, irrespective of delivery mode, demonstrated a significant discrimination between the novel and familiar arms, as indicated by the non-significant difference in time spent exploring these arms (Fig. 2A). Interestingly, at this stage, female CSD mice spent a significantly longer time in the novel arm compared to the familiar arm (Fig. 2A), but without changes in the number of entries (Fig. 2B). However, at PND55, both VD male and female mice spent more time exploring the novel arm than the familiar one (Fig. 2C,D), but this was not observed in the CSD groups. This suggests that VD mice, regardless of sex, exhibited intact spatial memory at PND55. In contrast, CSD appeared to impair spatial memory in both male and female mice.

**Fig. 3:**
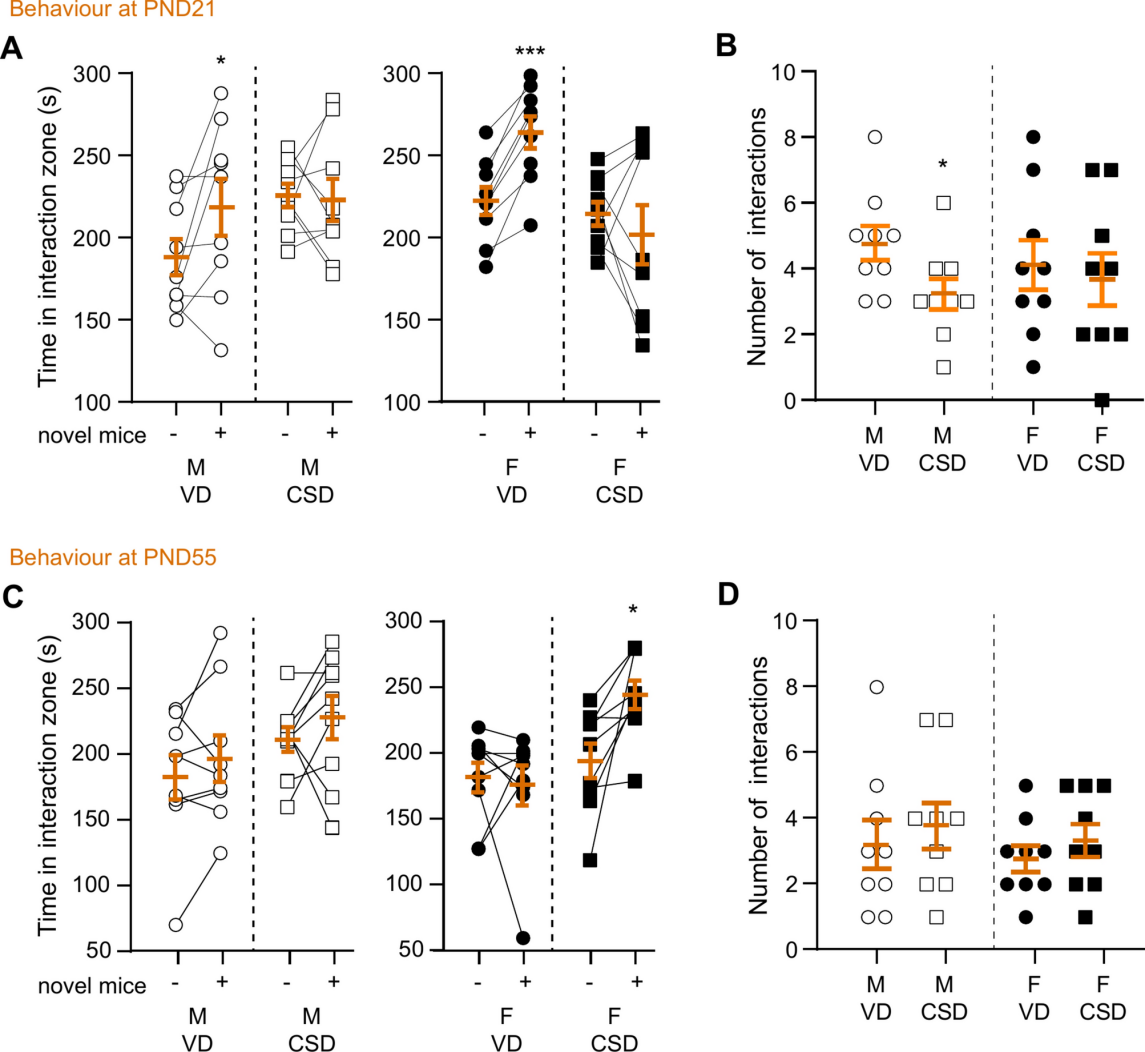
C-section delivery induces anxiety-like phenotypes. (**A**) Time spent in the interaction zone at PND21 when a novel mouse (‘novel mice’) of the same sex is present ( +) or absent (−). *p < 0.05 and ***p < 0.001, *versus* absence of target, paired t-test. (**B**) Total number of interactions performed when the target mouse is present. *p < 0.05, *versus* M VD, Welch’s unpaired t-test. (**C**, **D**) Similar assessments at PND55. (**C**) *p < 0.05, *versus* absence of target, paired t-test. M: males, F: females, VD: vaginal delivery, CSD: C-section delivery.

Next, we assessed social interaction in the different experimental groups under study (Fig. 3). At weaning, male and female VD mice displayed an interest for interacting with novel mice (Fig. 3A), assessed by a significant increased interaction time in the zone. However, CSD delivery impeded interaction, revealed by non-significant changes in interaction time in both male and female animals (Fig. 3A), indicative of impairment of social behavior in these animals^46,64^. These results were partially paralleled by significant decrease of the total number of interactions performed with the novel mice in the interacting zone, but only for males (Fig. 3B). At late adolescence, male and female VD mice did not spend significantly more time interacting with a novel mouse (Fig. 3C). Only CSD female mice presented increased interaction time when novel mice were introduced (Fig. 3C). Furthermore, total number of interactions remained stable across all 4 groups (Fig. 3D). Overall, these results suggest that CSD can impair social behaviour at weaning, but only in male mice.

The Y-maze test was used as we previously found that it is a good proxy to measure the effect of environmental stress on spatial memory in juvenile and adult mice^33,65–67^. At weaning, male and female VD mice were not able to discriminate between the familiar and novel arms. The onset of spontaneous recognition memory in juvenile mice can vary greatly^68^. Of note, as all mice litters are fostered by mothers which had delivered vaginally^43^, this cannot explain the absence of spatial recognition in the Y-maze test. In children, normative development of the hippocampus, a key brain structure to spatial memory, is different according to sex^69^. Such differences have also been reported in mice, together with the maturation of synaptic plasticity^70,71^. Children born via CSD display a delayed white matter development with microstructural alterations early in life^72,73^, which is a risk for neurodevelopmental disorders^74^. Nonetheless, the weaning process is known to be stressful for juveniles and we cannot rule out whether behavioural scores we observed were impacted by the stress of weaning^71,75^. The effect of CSD on adult male memory has been previously reported^76^. However, some discrepancies exist in the literature, as another study reports that CSD mice have altered memory at adolescence, but not adulthood^77^. The effect of CSD on memory has been linked to microbiota alteration^78^, however the effect of stress has not been studied and the causal effect of CSD-induced microbiota alteration and memory impairment remain to be established. Additional experiments are therefore needed to explore this possibility.

### Anxiety-like phenotypes induced by CSD

**Fig. 4:**
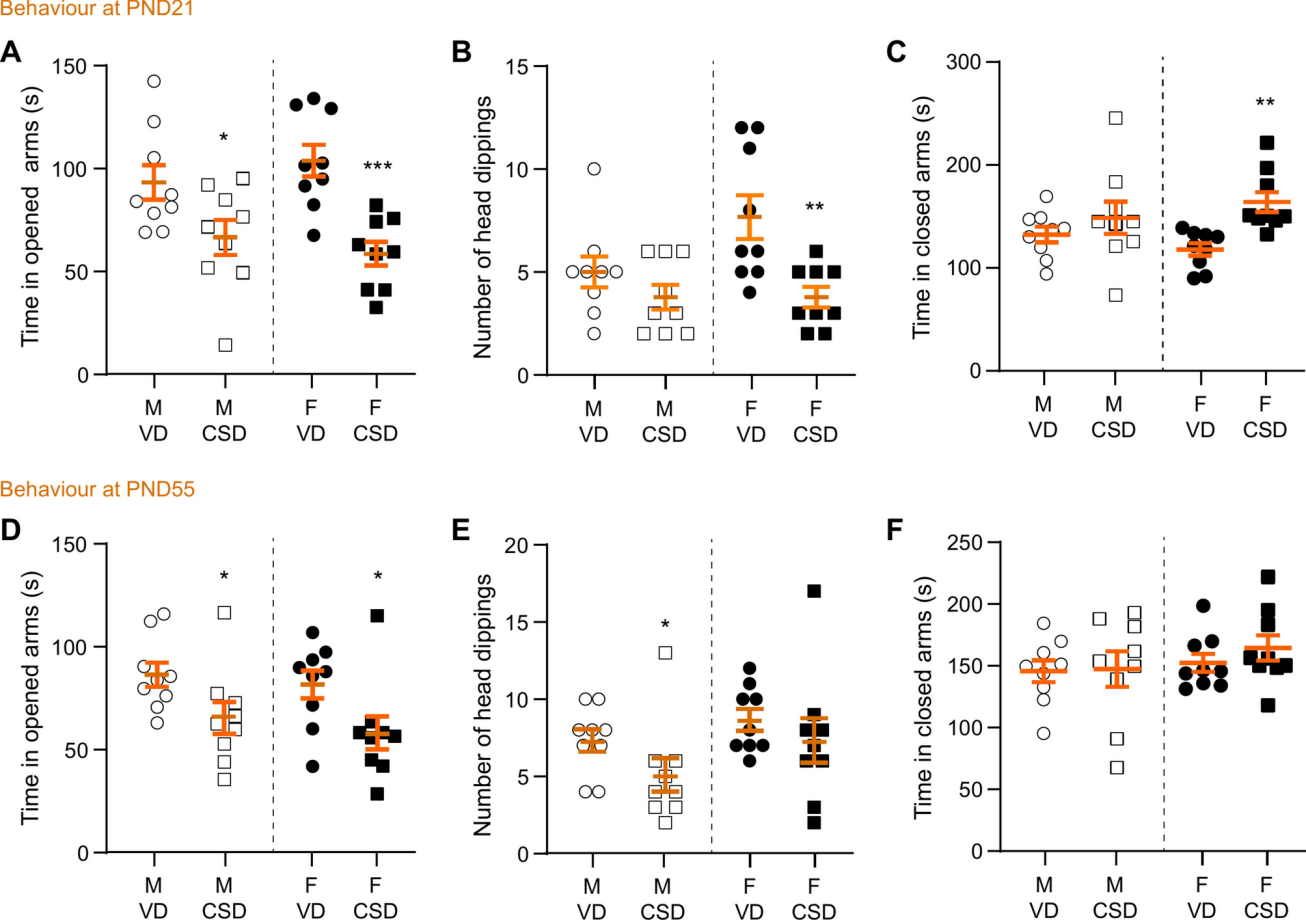
C-section delivery induces anxiety-like phenotypes. (**A**) Total time spent in the open arms during the elevated plus maze test at PND21. *p < 0.05 and ***p < 0.001, *versus* respective VD, paired t-tests. (**B**) Total number of head dippings. **p < 0.01, *versus* F VD, Welch’s unpaired t-test. (**C**) Time spent in the closed arms. **p < 0.01, *versus* F VD, Welch’s unpaired t-test. (**D**–**F**) Similar assessments at PND55. (**D**) *p < 0.05, *versus* respective VD, Welch’s unpaired t-test and Mann–Whitney test. (**E**) *p < 0.05, *versus* VD, Mann–Whitney test. M: males, F: females, VD: vaginal delivery, CSD: C-section delivery.

To further gathered insight on emotional behavior, we assessed anxiety-like behavior in male and female VD and CSD mice at weaning and late adolescence using the EPM test. The time spent by mice at weaning in the open arms of the EPM was significantly decreased in CSD mice, for both males and females (Fig. 4A). In addition, the total number of head dippings was significantly lower in CSD female mice as compared to VD female mice (Fig. 4B), an effect that was not observed in males. These observations were paralleled when measuring time spent in the closed arms (Fig. 4C). This suggests that CSD promotes anxiety-like phenotypes at weaning. At late adolescence, CSD mice presented significantly decreased time spent in the open arms, in both males and females (Fig. 4D), together with decreased number of head dippings, but only in males (Fig. 4E). There was no change in the time spent in the closed arms (Fig. 4F). Altogether, these results suggest that CSD induces anxiety-like behaviors that are present from weaning to later life.

**Fig. 5:**
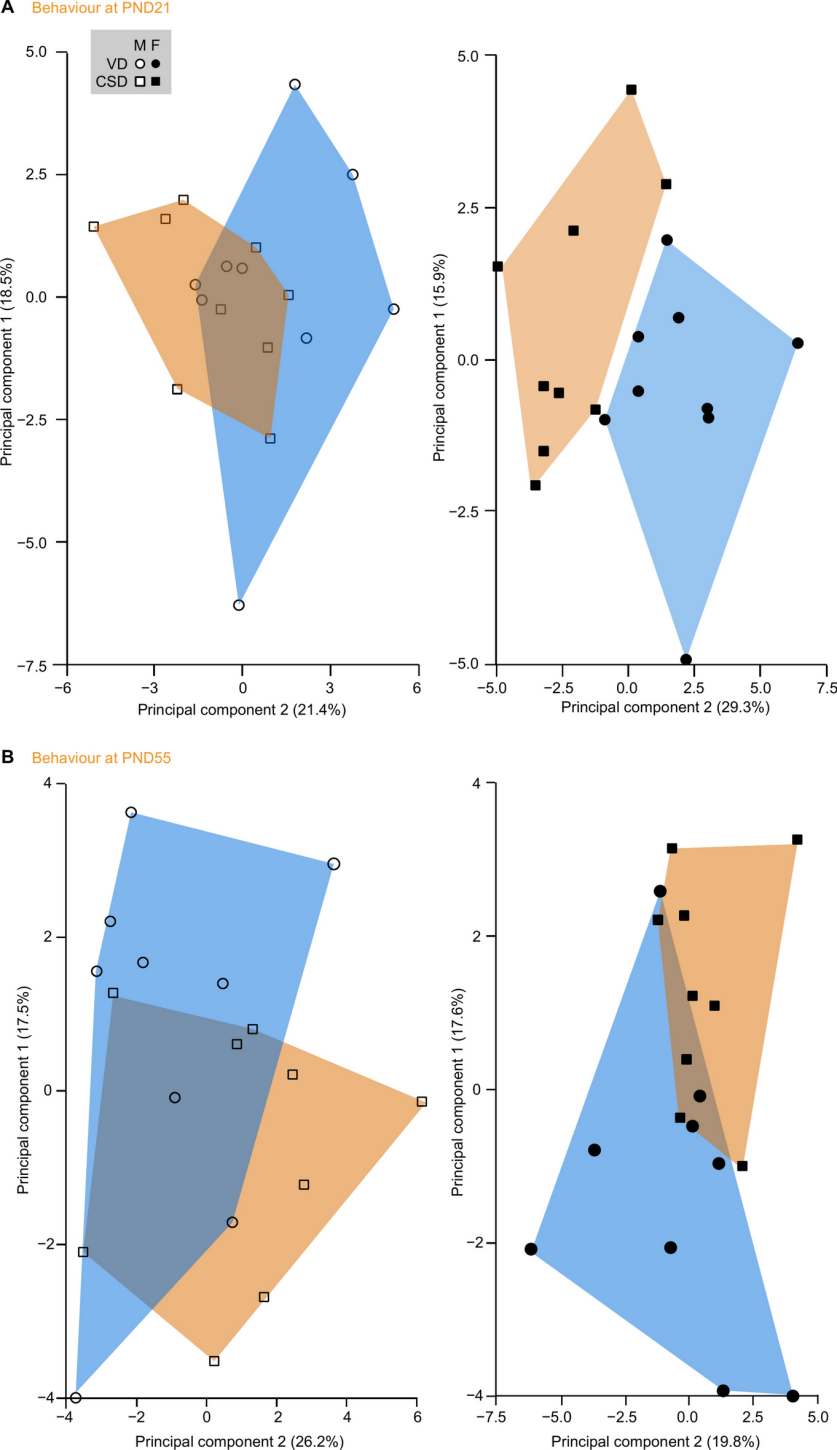
Segregation of mice based on behavioral results. Principal component analyses (PCA) were performed to test for clustering of animals based upon behavioral variables. (**A**) Successful clustering of females, but not males at weaning. Note that data variance capture by the two first components (1 and 2) was quite weak (39.9% and 45.2%, left and right graphs, respectively). (**B**) Weak clustering of females at late adolescence. Note that data variance capture by the two first components (1 and 2) was quite weak (43.7% and 37.4%, left and right graphs, respectively). M: males, F: females, VD: vaginal delivery, CSD: C-section delivery.

Finally, we examined the global behavioral profiles of mice at weaning or late adolescence, using 28 different behavioral parameters (listed in Supplementary Table S4). At weaning, we observed that CSD induces a behavioral segregation in females only (Fig. 5A), as animals born by CSD clustered away from those born by VD when data was projected onto the first two first principal component axes (the two axes that accounted for the highest data variability capture). At PND55, segregation of females by delivery mode was still observed (Fig. 5B), but to a lesser degree than at weaning. Overall, these observations suggest that females present anxiety phenotype when born by CSD, which might indicate that these mice could be at a higher risk to develop emotional behavior alterations, an effect that seems to persist during later life.

These results are aligned with previous observations in humans^47,79^ and rodents^76^, in which altered sociability is linked to CSD. However, sex differences have not been reported before. Using the elevated plus maze, we found that CSD mice spent less time exploring the open arm at weaning and late adolescence, regardless of sex, suggesting that overall CSD alters emotional behaviour towards an anxiety-like behaviour. Our observations are supported by previous work showing that emotional behaviour and stress axis are altered in adult male CSD mice^77,78^.

### CSD-induced neuroinflammation

**Fig. 6:**
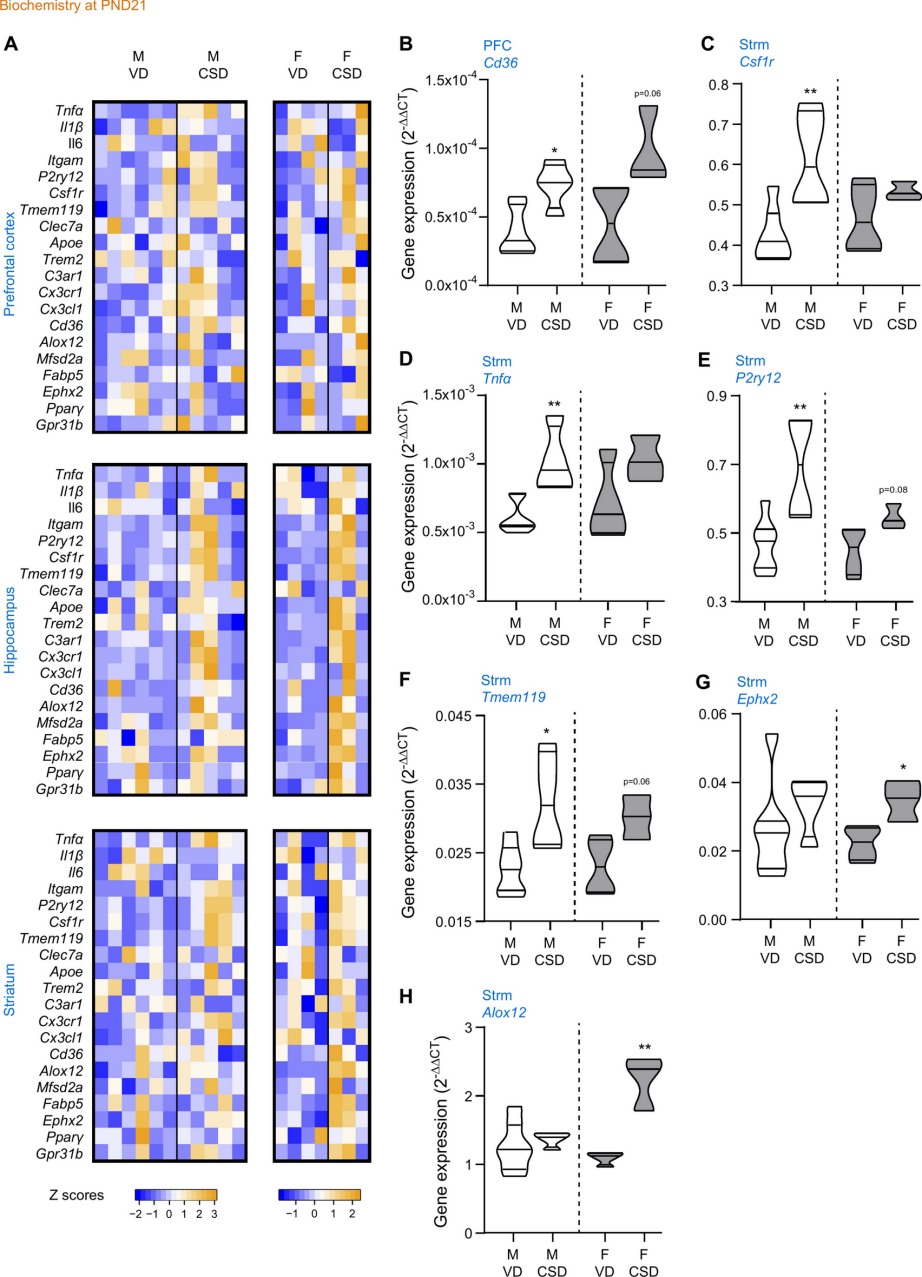
Metabolic shift induced by C-section delivery at weaning. Gene expression quantifications in different brain regions of mice: (**A**) Heatmaps of gene expression in the hippocampus, prefrontal cortex and striatum. (**B**) *Cd36* in PFC, (**C**) *Csf1r* in Strm, (**D**) *Tnfα* in Strm, (**E**) *P2ry12* in Strm, (**F**) *Tmem119* in Strm, (**G**) *Ephx2* in Strm, (**H**) *Alox12* in Strm. Abbreviated gene names were used, please see Supplementary Table S1 for further details. PFC: prefrontal cortex, HC: hippocampus, Strm: striatum. M: males, F: females, VD: vaginal delivery, CSD: C-section delivery. *p < 0.05 and **p < 0.01, *versus* respective CSD, unpaired t-tests (except in **D**, Mann–Whitney test).

**Fig. 7:**
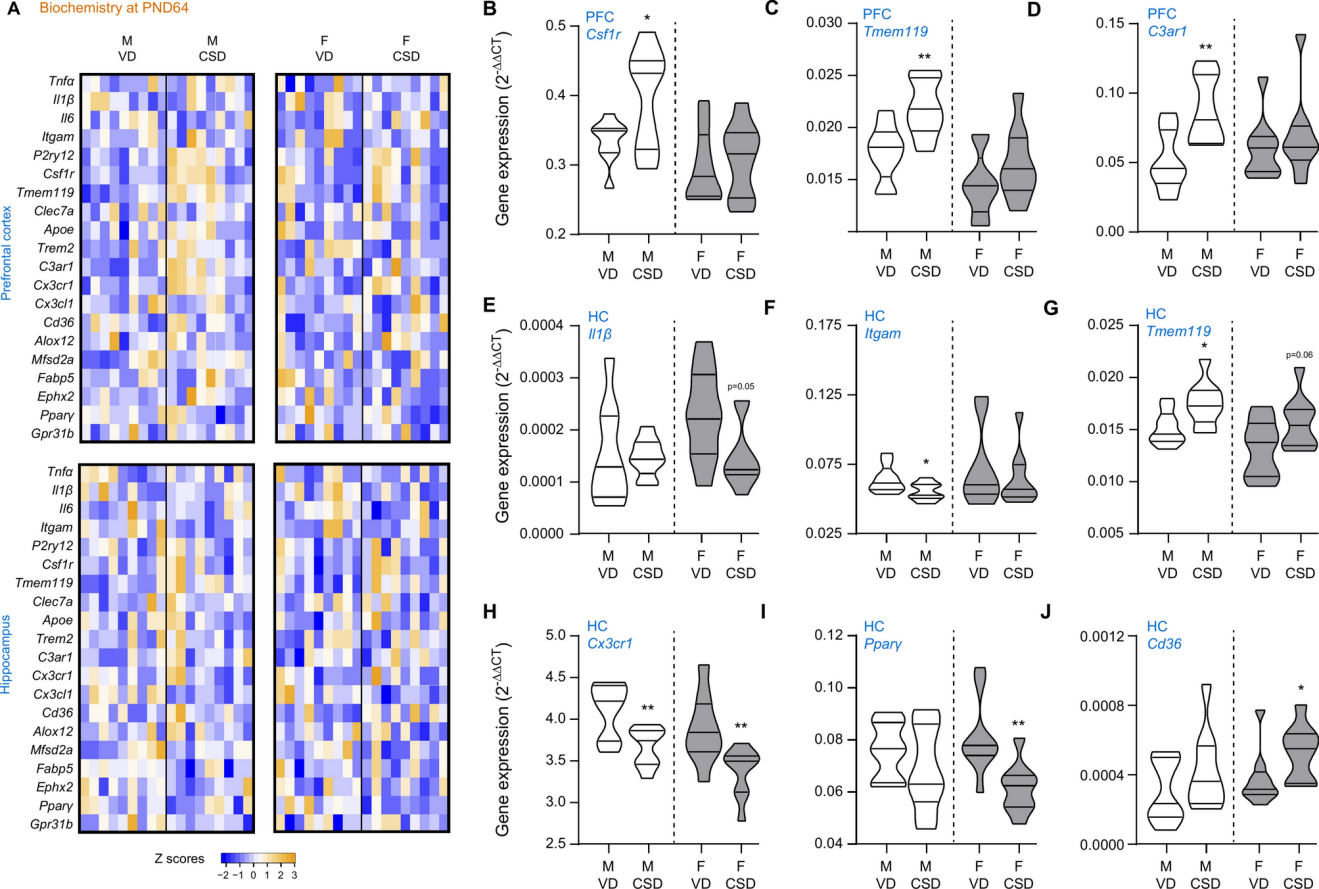
Metabolic shift induced by C-section delivery at late adolescence. Gene expression quantifications in different brain regions of mice: (**A**) Heatmaps of gene expression in the hippocampus and prefrontal cortex. (**B**–**D**) Quantification in the PFC for *Csf1r* (**B**), *Tmem119* (**C**) and *C3ar1* (**D**). (**E**–**J**) Quantification in the HC for *Il-1β* (**E**), *Itgam* (**F**), *Tmem119* (**G**), Cx3cr1 (**H**), *Pparγ* (**I**) and *Cd36* (**J**). Abbreviated gene names were used, please see Supplementary Table S1 for further details. PFC: prefrontal cortex, HC: hippocampus. M: males, F: females, VD: vaginal delivery, CSD: C-section delivery. *p < 0.05 and **p < 0.01, *versus* respective VD, unpaired t-tests (except in (**J**), Mann–Whitney test).

We then measured the expression of brain biomarkers of inflammation^80^, microglia phenotypes^25^ and lipid metabolism^49,81^ (Supplementary Table S1) in several brain structures at weaning (Fig. 6, cohort 1) and late adolescence (Fig. 7, cohort 2, which underwent behavioral analysis at PN21 and PND55 as presented in Fig. 1). These genes have been previously associated to memory and emotional behaviour impairment linked to environmental stress early in life^30,33,81,82^ and in neurodegenerative diseases^25^. Furthermore, they are also associated to memory performance^31,33,83^, anxiety-like behavior and social behavior associated to early-life environmental adversity triggered by stress^82,84^ or nutrition^49,82,84^. At weaning (PND21, Fig. 6A), a significant increase in mRNA gene expression of *Cd36* was measured in the prefrontal cortex (PFC) of CSD males (Fig. 6B), but not females. *Csf1r* mRNA expression was also significantly enhanced in the striatum (Strm) of CSD males (Fig. 6C). Furthermore, in the striatum of male CSD mice, *Tnfα* (Fig. 6D), *P2ry12* (Fig. 6E) and *Tmem119* (Fig. 6F) expressions were significantly increased. In females, no significant change in the expression of these five genes could be observed, although almost significant increases were observed for *Cd36* in the PFC (p = 0.06) and *Tmem119* in the striatum (p = 0.06). In contrast, significant increased expressions of *Ephx2* (Fig. 6G) and *Alox12* (Fig. 6H) were observed in females, an effect not observed in males. Overall, at weaning, microglia phenotype-related gene expression seems to be altered in males by CSD, in a brain region-independent manner. On the contrary, at weaning lipid metabolism-related genes are mostly impacted in females born via C-section and in a brain region-dependent manner.

At late adolescence (Fig. 7A), significant increased expressions of *Csf1r* (Fig. 7B), *Tmem119* (Fig. 7C) and *C3ar1* (Fig. 7D) were measured in the PFC of male CSD mice, which were not observed in female animals. Furthermore, a decrease of *IL-1β* expression was observed in the hippocampi of female mice (Fig. 7E) after CSD, whilst, in males, a significant decrease of *Itgam* was observed (Fig. 7F), together with a significant increase of *Tmem119* (Fig. 7G). A tendency of increased expression of *Tmem119* was observed in females after CSD, but this did not reach statistical significance (p = 0.06, Fig. 7G). In both males and females, CSD decreased the expression of *Cx3cr1* (Fig. 7H). *Pparγ* was found to be significantly decreased in female mice following CSD (Fig. 7I), whilst a significantly increased expression of *Cd36* was found (Fig. 7J). The expression of both *Pparγ* and *Cd36* remained unaffected by CSD in males.

Interestingly, *csfr1* and *tmem119* expression was still higher at late adolescence, together with those of *c3ar1*, which is involved in microglia phagocytosis^33^. Overall, these data suggest that in male, CSD triggers homeostatic and phagocytic microglia profiles^25,33^. Our results pinpoint some differences between male and female as at weaning, only genes (*alox12* and *ephx2*) related to the metabolism of oxylipins, which are polyunsaturated fatty acid (PUFA) derivatives with immune properties^81^, were upregulated in brain regions of female CSD mice. Changes in oxylipin profile could account for some alteration in brain wiring, as oxylipins have been shown to be key in microglia phagocytic activity of dendritic spines during brain development^33^. This effect seems to be transient as, at late adolescence, only *cx3cr1* and *cd36* mRNA expressions are respectively decreased and increased in the hippocampus of female CSD mice. Nevertheless, CSD is still affecting microglia-related genes at late adolescence. Whether the changes in immune and lipid metabolism gene expression in the brain is involved in behavioural impairment developed in CSD mice remains to be determined. To further get insight in the potential link between microglia profile and behavioural parameters, correlation analyses were performed and revealed that at late adolescence, *il-1β*, *clec7a* and *alox12* mRNA expression in the prefrontal cortex and *clec7a* in the hippocampus positively correlate to time spent in arm, a hallmark of anxiety. On the contrary, *mfsd2a* in the hippocampus and the prefrontal cortex is negatively correlated to anxiety. Il-1β and lipoxygenase12 (*alox12*) are involved in neuroinflammatory pathways, which are associated to stress response and anxiety^81,85^, while mfsd2a is a lysophosphatidylcholine co-transporter which is key to neurodevelopment and synapse maintenance^86,87^. The altered expression of these genes in several brain structures could indicate that CSD alters both lipid metabolism and neuroinflammatory pathways which are intricate^66,88^. Interestingly, *clec7a* is a marker of microglia associated to neurodegenerative diseases which also have a lipid metabolism signature^25,89^. In the developing brain, a subset of Arg1^+^ microglia with a high expression of *clec7a* and a low expression of homeostatic genes *P2ry12* and *Tmem119* have been identified as being phagocytic^90^. Whether the increased *clec7a* expression in the brain of CSD mice at weaning and late adolescence is a signature of an increase subset of phagocytic microglia remains to be determined. The maturation of microglia in the brain of CSD infants has been poorly addressed, however previous studies have revealed that CSD triggers gut barrier impairment and increase the susceptibility to inflammation^43,91^. We also report here a correlation between the expression of *cd36* and social behaviour, with the former potentially acting as the main driving force for neurodevelopmental trajectories, or due to a metabolic shift. Indeed, links between behaviour, *cd36* expression in the gut, the microbiota and inflammation were reported in previous studies^92,93^. CD36 has been reported as a potent marker of microglia phagocytic activity and is highly expressed in developing microglia^94^ . However, it is still unknown whether CSD-induced CD36 positive microglia is involved in social behaviour alteration.

### Correlations between behaviour and neuroinflammation

**Fig. 8:**
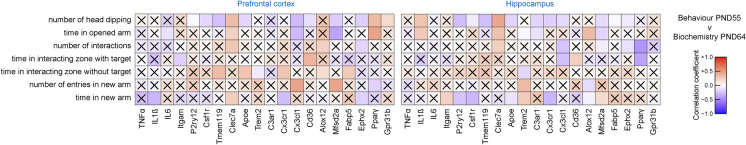
Correlations between behavior and biochemistry. Correlation matrices between behavioural scores at PND55 and gene expression at PND64 in the prefrontal cortex or hippocampus. Pearson’s coefficients. Crossed squares indicate non-significant coefficients.

**Fig. 9:**
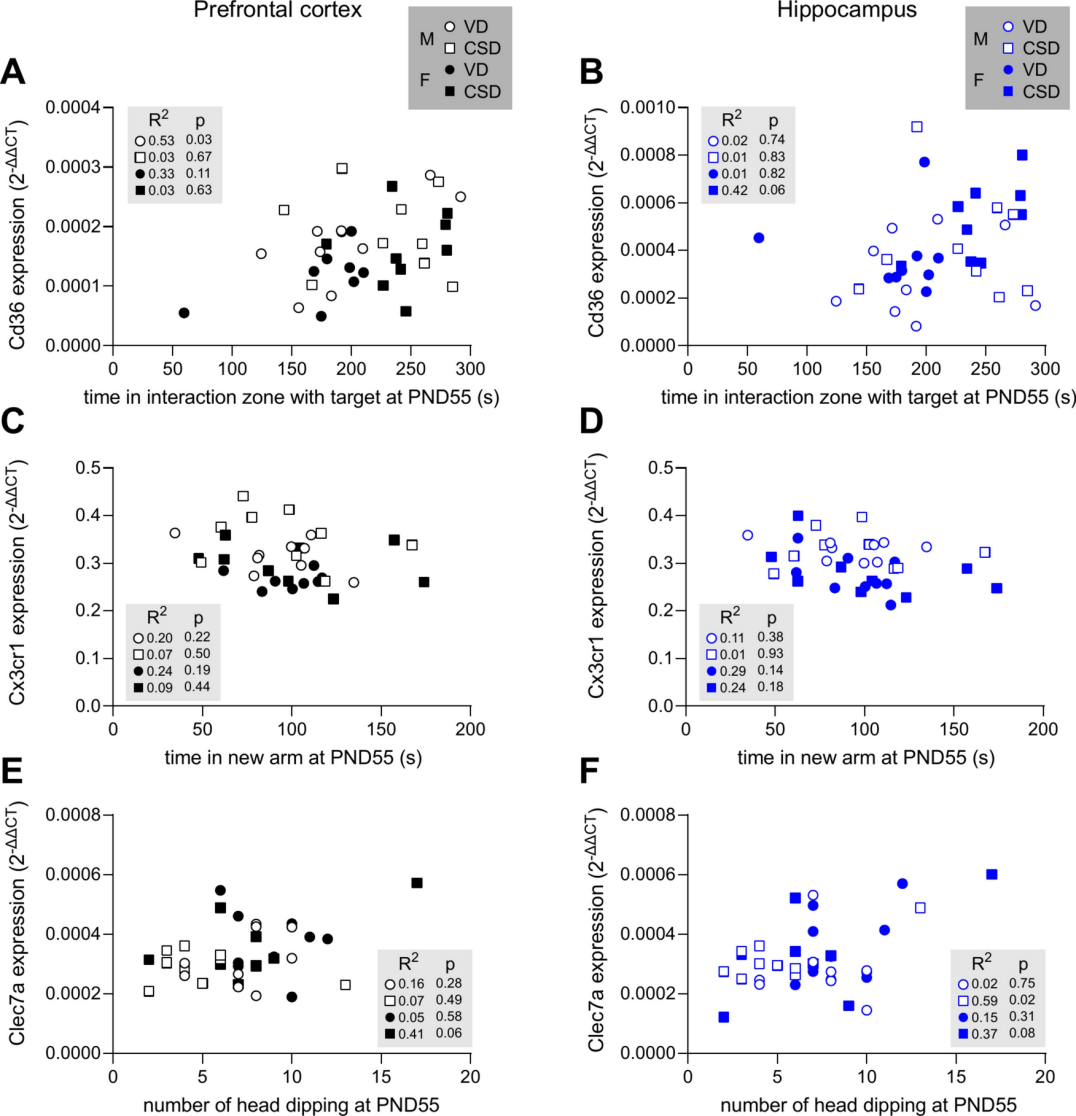
Correlations of interest. Correlations between expression of *Cd36* at PND64 in the prefrontal cortex (**A**) or hippocampus (**B**) and the time spent in the interaction zone at PND55 when a target mouse is present. Correlations between expression of *Cx3cr1* at PND64 in the prefrontal cortex (**C**) or hippocampus (**D**) and the time spent in the new arm at PND55. Correlations between expression of *Clec7a* at PND64 in the prefrontal cortex (**E**) or hippocampus (**F**) and the number of head dippings at PND55. R^2^ and *p* values are reported from Pearson’s correlation tests.

Next, we studied whether a linear correlation between behavioural profiles at PND55 and brain biochemical properties at PND64 could be established. To address this, we calculated Pearson’s correlation coefficients between behavior and brain biochemistry at late adolescence for aggregated animals (Fig. 8). We found a significant correlation between the time spent in interaction and the expression of *Cd36* in both the hippocampus and prefrontal cortex of all mice (Fig. 8). Indeed, a positive correlation emerged at PND55/PND64. Furthermore, *Cx3cr1* mRNA expression was negatively correlated to the time spent by mice in the new arm of the Y Maze (Fig. 8). Finally, a positive correlation between the expression of *Clec7a* and the number of head dippings performed in the EPM was observed at late adolescence (Fig. 8). All of these observations are detailed in Fig. 9 for individual groups, thus providing evidence for a link between microglia and lipid metabolism gene expression and altered behaviour in CSD mice, although only 2 significant correlations are observed when focusing on individual experimental groups (Fig. 9).

### Potential limitations

The number of animals used in each experimental groups of this study was heterogeneous, which was inherent to the proportion of sexes in the different cohorts (male *versus* female mice). Therefore, our results should be interpreted with caution, especially in the first cohort (PND21 animals). Whilst increasing the number of mice used in the present study would increase statistical power^95–97^, the number of animals used in research is strictly regulated and scrutinized by our local Ethics Committee and governmental bodies. We therefore decided to not generate additional litters, which is a potential limitation to our current observations.

In our second cohort (PND55/64 animals), the time between the first and second battery of behavioral tests could have influenced our subsequent biochemical analyses, as well as the repetition of these tests (at PND21 and PND55; second cohort). However, this might have been limited by including control groups (VD mice) in our experimental protocols, whilst also ensuring that the handling and housing conditions remained exactly the same between VD and CSD mice, throughout our study.

Surprisingly, we also noted that control animals (VD mice) not always performed adequately in some behavioural tests, regarding of sex. Indeed, control animals did not behave expectedly in some experiments (location memory at PND21, social interaction at PND55). These observations may be explained by external variables which were outside of our control, even if our housing facilities are limiting the impact of extrinsic variables. Neurobiological adaptation to stress is characterized by the expression of genes characteristic of neuroinflammation in the absence of disease, infection, or injury and is referred to “parainflammation”^98^. This long-term effect of stress could be linked to the crosstalk between neurons, immune cells and microglia^99,100^. In addition, tasks involving memory are known to induce short-term proinflammatory gene expression in the brain^101^, which is important for the retrieval performance^102^. In addition, parainflammation has a dual role in the brain^101^. In our experiments, both groups (VD and CSD) underwent the same behavioral and potential stressful experiences which could have influenced gene expression, highlighting that the delivery mode does indeed modify neuroimmune processes and emotional behaviour, with significant differences between VD and CSD mice.

In our experimental paradigm, we did not test whether some mice displayed dominant or submissive phenotypes after P21, which is a limitation to our current results. In fact, it is known that stress can greatly influence gene expression in the brain, especially genes related to neuroinflammation such as Il-6 and Il-1β^100^. Furthermore, hierarchy during housing of male mice has been shown to affect gene expression in the hippocampus and hypothalamus^103^.

Finally, we also noted some variation in the gestational day in VD groups. Indeed, birth occurred between gestational day 19 and gestational day 21 for VD mice. This could have influence our results, although this was observed in all experimental VD subgroups. We also ensured that all mice were adopted by another mother, regardless of sex or mode of delivery, in an attempt to limit potential bias related to maternal behaviour.

## Supplementary Information

Supplementary Information 1.

Supplementary Information 2.

Supplementary Information 3.

## Data Availability

Data used in the present study is available at FigShare using the following link: 10.6084/m9.figshare.25459183.v1.

## Abbreviations

ANOVA: Analysis of variation
ASD: Autism spectrum disorder
CSD: C-section delivery
E: Embryonic day
EPM: Elevated plus maze
F: Female
fam: Familiar
HC: Hippocampus
M: Male
NDDs: Neurodevelopmental disorders
nov: Novel
PFC: Prefrontal cortex
PND: Post-natal day
PUFA: Polyunsaturated fatty acid
SI: Social interaction
Strm: Striatum
VD: Vaginal delivery
