# Supplementary figures and images for: Modified neuroimmune processes and emotional behaviour in weaned and late adolescent male and female mice born via caesarean section
Source: Sci Rep. 2024 Nov 30;14:29807. doi: 10.1038/s41598-024-80770-y (PMC11608364; doi:10.1038/s41598-024-80770-y)

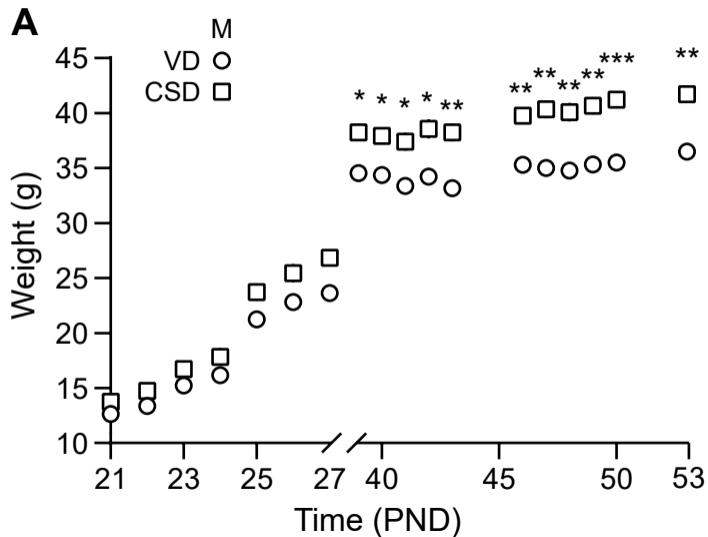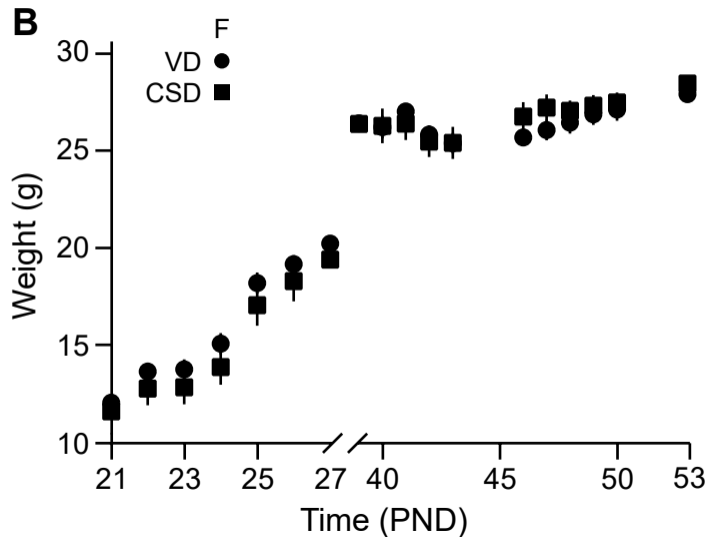

Supplement: Supplementary file 1 — Supplementary Information 1. [file 41598_2024_80770_MOESM1_ESM.pdf]
