# Supplementary material for: Modified neuroimmune processes and emotional behaviour in weaned and late adolescent male and female mice born via caesarean section
Source: Sci Rep. 2024 Nov 30;14:29807. doi: 10.1038/s41598-024-80770-y (PMC11608364; doi:10.1038/s41598-024-80770-y)
Supplement: Supplementary file 2 — Supplementary Information 2. [file 41598_2024_80770_MOESM2_ESM.docx]

**Supplementary Fig. S1 Body weight time-course.** Body weight curves in male (**A**) or female (**B**) mice. Note the non-linear abscissa between the 27^th^ and 39^th^ PND. * p<0.05, ** p<0.01 and ***p<0.001, *versus* VD, Šidák’s *post-hoc* tests after significant two-way repeated measures ANOVA. Note that SEM are displayed, but may be smaller than symbol size.

**Supplementary Table S1**. Genes of interest in the present study. Genes that are listed in this table but do not appear in the main manuscript were found to be unaltered by birth mode or sex (no change in expression assessed by qPCR).

| Gene code | Gene name | Classification |
| --- | --- | --- |
| TNFα | Tumor necrosis factor alpha | Chemokine |
| IL-1β | Interleukine 1 beta | Chemokine |
| IL-6 | Interleukine 6 | Chemokine |
| Itgam | Integrin alpha M chain | Membrane protein |
| P2ry12 | Purinergic receptor P2Y, G-protein coupled 12 | Membrane receptor |
| Csf1r | Colony stimulating factor 1 receptor | Membrane receptor |
| Tmem119 | Transmembrane protein 119 | Membrane receptor |
| Clec7a | C-type lectin domain containing 7A | Membrane receptor |
| Apoe | Apolipoprotein E | Enzyme |
| Trem2 | Triggering receptor expressed on myeloid cells 2 | Membrane receptor |
| C3ar1 | Complement C3a receptor 1 | Membrane receptor |
| Cx3cr1 | C-X3-C motif chemokine receptor 1 | Membrane receptor |
| Cx3cl1 | C-X3-C motif chemokine ligand 1 | Chemokine |
| Cd36 | CD36 molecule | Membrane receptor |
| Alox12 | Arachidonate 12-lipoxygenase | Enzyme |
| Mfsd2a | MFSD2 lysolipid transporter A, lysophospholipid | Membrane protein |
| Fabp5 | Fatty acid binding protein 5 | Cytoplasmic protein |
| Ephx2 | Epoxide hydrolase 2 | Enzyme |
| Pparγ | Peroxisome proliferator activated receptor gamma | Nuclear receptor |
| Gpr31b | G protein-coupled receptor 31 | Membrane protein |

**Supplementary Table S2**. RT-qPCR primer sequences.

| Gene | TaqMan primer | SYBR Green | |
| --- | --- | --- | --- |
|  |  | Forward | Reverse |
| TNFα | Mm00443258_m1 |  |  |
| IL-1β | Mm00434228_m1 |  |  |
| IL-6 | Mm00446190_m1 |  |  |
| Itgam |  | CTCATCACTGCTGGCCTATACAA | GCAGCTTCATTCATCATGTCCTT |
| P2ry12 | Mm00446026_m1 |  |  |
| Csf1r | Mm01266652_m1 |  |  |
| Tmem119 | Mm00525305_m1 |  |  |
| Clec7a | Mm01183349_m1 |  |  |
| Apoe | Mm00437573_m1 |  |  |
| Trem2 | Mm04209422_m1 |  |  |
| C3ar1 |  | GATTTGTTGGTGGCTCGCAG | TTGGGGTTGAAACAGAGGCC |
| Cx3cr1 | Mm00438354_m1 |  |  |
| Cx3cl1 | Mm00436454_m1 |  |  |
| Cd36 | Mm00432403_m1 |  |  |
| Alox12 |  | CGCCTGCAGCCAGACATGGTA | GGCAGGTCAAGTTGCTCATTCCG |
| Mfsd2a |  | ATGCTCTCGGCCACATTCAC | ATGAGGGCCACCAAGATGAG |
| Fabp5 | Mm00783731_s1 |  |  |
| Ephx2 |  | GATCCGCCTGCATTTTGTGG | AGAACACGAAAGCCTGCCTG |
| Pparγ |  | TCAGAAGTGCCTTGCTGTGG | ACAGCTTCTCCTTCTCGGC |
| Gpr31b |  | AGTGTGTGCTGGGCCTTATG | GCAGCAAAGAATGGCAGACTG |

**Supplementary Table S3**. Statistical analyses performed in the current study. Normal distributions were assessed via Shapiro-Wilk tests. Df: degree of freedom. ANOVA: analysis of variation, RM: repeated measures. p values are encoded as following: * p<0.05, **p<0.01 and ***p<0.001. M: males, F: females, VD: vaginal delivery, CSD: C-section delivery.

- *Please note that this table is supplied as a separate spreadsheet file.*

**Supplementary Table S4**. Variables used for behavioral analysis using principal component analysis (PCA). s: seconds, au: arbitral units. n values represent numbers.

| Behavioral test | Variable used for PCA | Unit |
| --- | --- | --- |
| Y maze test | Total time spent in the familiar arm | s |
|  | Total time spent in the new arm | s |
|  | Total distance in the familiar arm | au |
|  | Total distance in the new arm | au |
|  | Entries in the familiar arm | n |
|  | Entries in the new arm | n |
|  | Maximum speed in the familiar arm | au |
|  | Maximum speed in the new arm | au |
| Social interaction test | Total time spent in the safe zone without target | s |
|  | Total time spent in the safe zone with target | s |
|  | Total time spent in the corner zones without target | s |
|  | Total time spent in the corner zones with target | s |
|  | Total time spent in the interaction zone without target | s |
|  | Total time spent in the interaction zone with target | s |
|  | Total distance in the safe zone without target | au |
|  | Total distance in the safe zone with target | au |
|  | Total distance in the corner zones without target | au |
|  | Total distance in the corner zones with target | au |
|  | Total distance in the interaction zone without target | au |
|  | Total distance in the interaction zone with target | au |
|  | Total number of interactions with the target | n |
| Elevated plus maze test | Total time spent in the closed arms | s |
|  | Total time spent in the opened arms | s |
|  | Total time spent in the central zone | s |
|  | Total distance in the closed arms | au |
|  | Total distance in the opened arms | au |
|  | Total distance in the central zone | au |
|  | Head dippings | n |
